# Childhood and Adulthood Predictors of Community Participation by Autistic Adults With and Without Intellectual Disability

**DOI:** 10.1111/jar.70001

**Published:** 2024-12-23

**Authors:** Lauren A. Cameron, Bruce J. Tonge, Patricia Howlin, Stewart L. Einfeld, Roger J. Stancliffe, Kylie M. Gray

**Affiliations:** ^1^ Centre for Developmental Psychiatry and Psychology, Department of Psychiatry, School of Clinical Sciences at Monash Health Monash University Clayton Australia; ^2^ School of Psychology Deakin University Burwood Australia; ^3^ Centre for Research in Intellectual and Developmental Disabilities University of Warwick Coventry UK; ^4^ Institute of Psychiatry, Psychology and Neuroscience King's College London London UK; ^5^ Brain and Mind Centre University of Sydney Sydney Australia; ^6^ University of Sydney Centre for Disability Research and Policy Sydney Australia; ^7^ School of Social Policy and Society University of Birmingham Birmingham UK

**Keywords:** autism, community participation, intellectual disability, longitudinal, predictors

## Abstract

**Background:**

Few studies have explored community participation for autistic adults, with or without intellectual disability. This study aims to investigate how autistic adults participate in the community, and the childhood and adulthood factors that predict community participation in adulthood.

**Method:**

Eighty‐four autistic adults (mean age 34 years; 67% with co‐occurring intellectual disability) initially recruited as children and adolescents, participated in the current study. Community participation frequency and variety were measured in adulthood. Childhood and adulthood predictors of community participation were investigated.

**Results:**

Participants engaged in the community an average of 18.2 times (range 0–49) over the previous 30‐day period, in an average of 6.3 different activities (range 0–13). Childhood and adulthood factors (autism symptoms, intellectual disability, living arrangements) were associated with community participation.

**Conclusion:**

Areas for additional support and resources were identified.

## 1 Introduction

Community participation, including participation in informal groups, social clubs, common interest groups, and recreational and leisure activities, has been identified by the World Health Organisation as important to the health and functioning of all people (World Health Organisation 2001). Further, the benefits of community participation for children, adolescents and adults with varying intellectual, developmental and physical disabilities have been well documented, including greater social inclusion and friendships, independence and improved physical and mental wellbeing (Andrews, Falkmer, and Girdler 2015). Despite the known benefits, community participation for individuals with intellectual and developmental disabilities, including Autism Spectrum Disorder (hereafter referred to as autism), can be limited and challenging. Systematic reviews have consistently highlighted lower rates of community participation by children and adults with autism^1^ and intellectual disability compared with not autistic peers (Askari et al. 2015; Cameron et al. 2021; Verdonschot et al. 2009). In addition, rates of community participation decline over time from adolescence to adulthood (Myers et al. 2015), with adults participating in significantly fewer community activities and at a significantly lower frequency than the wider population (Shea et al. 2021).

While participation overall is more limited, the type of participation in community, recreation and leisure activities for individuals with autism is important to consider. For example, adolescents and young adults with autism may participate significantly more in solo leisure activities (e.g. going for a walk, engaging in a hobby) and organised group activities (e.g., special interest groups) than their not autistic peers, while participation in community activities (e.g., going to a café, attending sports events) may be more limited (Gray et al. 2014; Orsmond, Krauss, and Seltzer 2004). More recently, research has demonstrated that autistic adults often participate in online social or recreational activities, such as solo videogames or online multi‐player role playing games (Cameron et al. 2021), suggesting that, while engagement in the wider community might be limited, adults are engaging and participating in activities with like‐minded people, building their relationships and sharing interests with others. A recent study found that adults reported engaging in activities that support their daily living, such as shopping and running errands, as well as more social activities, such as going out to restaurants, or visiting with family and friends, to be important to them (Shea et al. 2021).

Many factors are likely to influence participation in the community, although few studies have explored these. Personal factors, such as anxiety and mental health problems, communication and social skill difficulties, and lack of necessary planning skills, as well as environmental factors, such as living arrangements, household income, access to services, have been identified as potential barriers to community participation (Cameron et al. 2021; Myers et al. 2015; Shea et al. 2021; Song et al. 2022). Further, better conversational ability, daily living and functional skills, and access to transport, resources and opportunities have been associated with higher rates of community participation in some studies (Cameron et al. 2021; Chan et al. 2021). However, research to date has largely focused on younger adults and individuals without intellectual disability. Additional research is needed to understand how these factors may impact a broader range of adults.

Inclusion of autistic adults with co‐occurring intellectual disability is lacking in the literature. A recent systematic review reported that only a third of research studies exploring community participation included adults with co‐occurring intellectual disability (Cameron et al. 2021). This is particularly concerning, as individuals with co‐occurring intellectual disability will have different needs and support requirements, as well as different goals in relation to community participation, than individuals with autism alone. Shea et al. (2021) reported differences in how autistic adults with and without co‐occurring intellectual disability rated the importance and recent participation of various activities, for example, going shopping or running errands. These differing needs are further evident in the types of interventions that are effective in supporting autistic individuals and individuals with intellectual disability to enhance their social and community participation (Giummarra, Randjelovic, and O'Brien 2022). It is important for research to ensure that all autistic adults are represented when seeking to understand community participation support experiences, needs and resources.

This study aimed to explore how adults with autism participate in the community, in particular, how often, and in how many different activities. This study also aimed to evaluate the childhood and adulthood factors associated with community participation outcomes in adulthood. Childhood factors included degree of intellectual disability, behaviour and emotional problems, autism symptoms and socioeconomic disadvantage. Factors in adulthood included degree of intellectual disability, behaviour and emotional problems, autism symptoms, socioeconomic disadvantage, living arrangements and daytime activities.

## 2 Method

### 2.1 Sample

Participants were part of the Australian Child to Adult Development (ACAD) Study and were recruited from metropolitan and regional areas of Victoria and New South Wales, Australia in 1991 through health and education agencies (Einfeld and Tonge 1996a, 1996b). Data were collected at six time points: Time 1 (1991–1993), Time 2 (1995–1996), Time 3 (1999), Time 4 (2002–2003), Time 5 (2007–2009) and Time 6 (2016–2019). Time 1 and Time 6 are the focus for the current study.

At entry to the study, participants were likely to be representative of children in the community who had an autism diagnosis and were receiving services (Tonge and Einfeld 2003). All participants met criteria for DSM‐III‐R Autistic Disorder (American Psychiatric Association 1987) following clinical assessment by a multidisciplinary team at study entry. Participants were reassessed at Time 2 to confirm diagnosis against DSM‐IV criteria (American Psychiatric Association 1994; Gray et al. 2012). At Time 6, diagnoses were reviewed again, using all clinical information gathered in the study, including the Autism Diagnostic Interview‐Revised (Rutter, Le Couteur, and Lord 2003). All Time 6 participants met current DSM‐5 criteria for Autism Spectrum Disorder (American Psychiatric Association 2013).

At Time 1 there were 119 participants. Participants were mostly male (*n* = 98, 82%) and were aged between 2.8 and 19.8 years old. A total of 84 adults with autism participated at Time 6 (response rate 75%). Participants were aged between 26.8 and 44.2 years (*M* = 34.2, SD = 4.5) and 81% (*n* = 68) were male. See Table 1 for further details of the sample, including, age, gender and degree of intellectual disability at Time 1 and Time 6. There were no significant differences between those who participated at Time 6 and those who did not in terms of degree of intellectual disability, *χ* ^2^ (4, *n* = 119) = 0.05, *p* = 0.97, or behaviour and emotional problems at Time 1 (DBC2 mean item score), *t*(117) = 1.50, *p* = 0.14.

**TABLE 1:** Sample demographics and descriptive statistics (*M*, SD) of behaviour and emotional problems and autism symptoms at Time 1 and Time 6.

	Time 1, *n* = 119	Time 6, *n* = 84
Male (%)	98 (82%)	68 (81%)
Female (%)	21 (18%)	16 (19%)
Mean age (SD)	8.7 (4.3)	34.2 (4.5)
Age range (years)	2.8–19.8	26.8–44.2
Degree of intellectual disability (*n*, %)		
Average	11 (9%)	14 (17%)
Borderline	16 (13%)	13 (16%)
Mild	29 (24%)	13 (16%)
Moderate	46 (39%)	21 (25%)
Severe/profound	17 (14%)	23 (27%)
Socioeconomic disadvantage		
Mean IRSD (SD)	1025 (58.9)	998.25 (68.7)
IRSD range	902–1179	817–1126
DBC2 TBPS MIS^a^	0.64 (0.26)	0.47 (0.27)
Mental health disorder diagnosis^b^ (*n*, %)	—	54 (68%)
Autism symptoms (*M*, SD)		
DBC2‐P Autism Screening Algorithm	27.45 (10.87)	—
ADI‐R Social/Communication^c^	—	2.39 (1.02)
ADI‐R‐Restricted and Repetitive Behaviours^c^	—	0.64 (0.39)

Abbreviations: ADI‐R = Autism Diagnostic Interview‐Revised; DBC2 TBPS = Developmental Behaviour Checklist 2 Total Behaviour Problem Score; DBC2‐P = Developmental Behaviour Checklist 2—Parent; IRSD = Index of Relative Socioeconomic Disadvantage; MIS = mean item score.

^a^ Time 6: *n* = 75.

^b^ *n* = 79.

^c^ *n* = 69.

### 2.2 Measures

Demographic information, including age, gender, socioeconomic disadvantage, current living arrangements and daytime activity was collected via parent/carer questionnaire at Time 1 and by parent/carer and/or self‐report questionnaire at Time 6 of this study. Living arrangements were categorised as living independently, living with family or living in supported accommodation. Daytime activity was categorised as no activity, disability specific activity or mainstream employment or education.

#### 2.2.1 Degree of Intellectual Disability

At Time 1, participants were grouped according to their degree of intellectual disability: no intellectual disability, mild, moderate or severe degree of intellectual disability (Gray et al. 2014). At Time 6, current degree of intellectual disability was reviewed and classified by two authors (L.A.C. and K.M.G.) following DSM‐IV (American Psychiatric Association 1994) and DSM‐5 (American Psychiatric Association 2013) criteria. Classification was based on a range of assessment information, including scores on standardised assessments of cognitive and adaptive skills in addition to qualitative review of daily functioning. Specific domains assessed included adaptive behaviour (Adaptive Behaviour Assessment System; Harrison and Oakland 2015), daily living skills (Index of Social Competence; McConkey and Walsh 1982) and cognitive assessment (Wechsler Abbreviated Scale of Intelligence; Wechsler 2011).

#### 2.2.2 Behaviour and Emotional Problems

The Developmental Behaviour Checklist 2 (DBC2) is an informant‐report measure of behaviour and emotional problems in individuals with intellectual and developmental disabilities (Gray et al. 2018). Respondents are asked to rate each item on a three‐point scale ranging from 0 (*not true as far as you know*) to 2 (*very true or often true*), based on the previous 6 months. The DBC2 produces a Total Behaviour Problem Score (TBPS) as well as scores across five subscales. Two versions of the DBC2 have been used across the duration of this study; the DBC‐Parent/carer report (DBC2‐P; Time 1) and the DBC‐Adult (DBC2‐A; Time 6). Subscales for the DBC2‐P include *Disruptive*, *Communication Disturbance, Anxiety, Self‐Absorbed*, and *Social Relating*. For the DBC2‐A, subscales include *Disruptive, Communication and Anxiety Disturbance, Depressive, Self‐Absorbed*, and *Social Relating*. Mean item scores (MIS) were calculated for all subscales at Time 1 and Time 6 (Taffe et al. 2008). The DBC2‐P was completed by parents/carers at Time 1 and the DBC2‐A completed by parents or carers at Time 6. The DBC2 has well established psychometric properties (Einfeld and Tonge 2002; Gray et al. 2018; Mohr, Tonge, and Einfeld 2005; Mohr et al. 2011).

#### 2.2.3 Autism Symptoms

The Developmental Behaviour Checklist 2 Autism Screening Algorithm (DBC2 ASA; Brereton et al. 2002) was used as a measure of childhood (Time 1) autism symptoms. It is calculated from the DBC2‐P and has established validity as a screening tool for autism (Brereton et al. 2002; Steinhausen and Metzke 2004).

Autism symptoms at Time 6 were assessed using the Autism Diagnostic Interview‐Revised (ADI‐R; Rutter, Le Couteur, and Lord 2003), completed with parents or carers. For this study, only the current algorithm ADI‐R domain scores were used as a measure of current autism symptoms. Due to challenges of recall, and as a number of interviews were completed with professional carers and not parents, only current item codes were used in analysis. The ADI‐R algorithm produces scores on three domains: Qualitative Abnormalities in Reciprocal Social Interaction (hereafter referred to as the Social domain), Qualitative Abnormalities in Communication (hereafter referred to as the Communication domain) and Restricted, Repetitive and Stereotyped Patterns of Behaviour (hereafter referred to as the Restricted and Repetitive Behaviour domain). For the purposes of this study, mean algorithm scores were calculated for each of the ADI‐R domains, due to different numbers of items contributing to the Communication domain algorithm score depending on the verbal ability of the individual. Mean scores, therefore, allowed for a more consistent approach across analyses. The Social and Communication domains were also highly correlated (*r* = 0.72), and were therefore combined to create an overall Social/Communication domain score. Current ADI‐R interviews were missing for *n* = 9 participants, and current domain algorithm scores were unable to be calculated for *n* = 6 participants due to missing information.

#### 2.2.4 Mental Health

Interviews were conducted with parents or carers, as well as the participant themselves where possible, to determine presence of any mental health problems at Time 6. Symptom level information was gathered using the Structured Clinical Interview for DSM‐5, research version (SCID‐5; First et al. 2015). Mental health diagnoses were determined by clinical case reviews with a panel of the authors (K.M.G., B.J.T., P.H., S.L.E.), experts in the field of intellectual and developmental disabilities and mental health. Participants were categorised according to whether they had a current mental health disorder diagnosis or not.

#### 2.2.5 Socioeconomic Disadvantage

The Socio‐Economic Index for Areas (SEIFA; Australian Bureau of Statistics 2016) was used as a measure of socioeconomic disadvantage. SEIFA produces a ranking of areas in Australia based on relative socioeconomic advantage and disadvantage. The Index of Relative Socioeconomic Disadvantage (IRSD) was calculated for each participant based on where they were living at Time 1 and at Time 6. A lower score indicates relatively greater disadvantage.

#### 2.2.6 Community Participation

The Index of Community Involvement‐Revised (ICI; Raynes, Sumpton, and Pettipher 1989) is a 16‐item scale that assesses the frequency and variety of participation in social, community and leisure activities over the previous month, completed as an informant or self‐report measure. At Time 6, the ICI was completed by parents or carers, as well as by the participant themselves where possible. Self‐report responses were used in the first instance where they were available. Each item was rated based on how often each activity was participated in over the previous month on a 6‐point scale, ranging from 0 (*no participation*) to 5 (5 or more times). Respondents were also asked to indicate whether the activity was usually undertaken independently, or with support from parents or professional carers. *Frequency* was scored by summing the total of all items (range 0–80), indicating how often or how many times the participant engaged in community activities. *Variety* was scored by calculating the number of activities that have been participated in at least once over the previous month (range 0–16), indicating the range and variety of activities the participant engaged in.

### 2.3 Procedure

All participants were invited to participate at each time point and were sent a questionnaire to be completed by a parent or carer, and, at Time 6, the adult themselves where possible. At Time 6, the initial contact to invite participants to participate in the current study was with the parent/carer that provided consent for participation at the previous timepoint. During a phone call with the research team, current guardianship arrangements for the adult was determined. Where the parent or carer was the legal guardian, they provided consent and were also asked whether the adult was able to provide their own consent and complete a self‐report questionnaire and/or interview. Where adults were their own legal guardians and/or the parent deemed they had capacity, they provided their own consent. At Time 6, interviews were also conducted with parents/carers and the adult participant. Ethics approval was obtained from Monash University Human Research Ethics Committee (CF15/1045‐2015000486).

### 2.4 Statistical Analysis

Descriptive statistics were calculated for all variables. Associations between predictor variables and community participation outcomes were assessed using Pearson's correlations, independent samples *t*‐test or one‐way analysis of variance as appropriate, in order to determine variables to enter into regression models. The strength of the association as per Cohen (1988), as well as statistical significance, was considered in this determination. Regression analyses were performed with the determined predictor variables. Correlational analyses were conducted between all predictor variables to identify multicollinearity. No multicollinearity was detected between predictor variables entered into each regression model. Significance was set at *p* < 0.05 for interpretation of regression models.

At Time 6, there were missing data across a number of predictor variables (ABAS [*n =* 5 missing], ADI‐R [*n =* 9 missing], DBC2‐A [*n =* 9 missing]). To maximise sample size and reduce overfitting in regression models, mean algorithm scores for each of the ADI‐R domains (Social/Communication, and Restricted and Repetitive Behaviours) were assessed in a separate regression model.

## 3 Results

### 3.1 Community Participation at Time 6

The ICI was completed for 82 participants at Time 6 (*n* = 52 parent/carer report, *n* = 30 self‐report). Over the previous 30‐day period, participants engaged in community activities an average of 18.2 times (SD = 9.6; range 0 to 49). An average of 6.3 (SD = 2.8) different activities were participated in, ranging from 0 to 13. Two participants had not participated in any activities over the past 30 days. There was a large correlation between frequency and variety (*r* = 0.85, *p* < 0.001), demonstrating that a greater variety of activities was associated with increased frequency of participation.

The number of participants that had engaged in each ICI activity over the previous month is provided in Table 2. Only four activities were participated in by more than half of the sample; shopping (*n* = 70; 85%), café/restaurant (*n* = 63; 77%), trips out with family/friends (*n* = 61; 74%) and going on a bus (*n* = 43; 52%). Less than a quarter of participants had been to more structured community events, such as sports events (*n* = 20; 24%), cinemas (*n* = 20; 24%), concert/play (*n* = 10; 12%) or church services (*n* = 8; 10%).

**TABLE 2:** Frequency of participation in each Index of Community Involvement activity (*N* = 82).

Index of Community Involvement items	*n* (%)
Been shopping	70 (85%)
Been to a café/restaurant	63 (77%)
Had trips out with family/friends	61 (74%)
Been on a bus	43 (52%)
Been on a holiday (past 12 months)	41 (50%)
Been to a hairdresser	36 (44%)
Been to a hotel/pub/bar	32 (39%)
Been to a bank	32 (39%)
Been to a social club	28 (34%)
Been on an overnight stay to family/friends	25 (30%)
Had family or friends in for a meal	23 (28%)
Been to a sports event	20 (24%)
Been to a cinema	20 (24%)
Been to a concert/play	10 (12%)
Been to church	8 (10%)
Had guests to stay	8 (10%)

For each activity participated in, respondents were asked whether the activity was usually undertaken independently or with parents/carers. For all activities, except for *had guests to stay*, and *been to a church*, participants overwhelmingly participated with a parent or carer rather than independently. Overall, 14 participants (17.5%) participated in all activities independently, 26 (32.5%) participated in a combination of activities both independently and with a parent/carer, and 40 (50%) always participated in activities with the support of a parent/carer.

### 3.2 Associations Between Time 1 (Childhood) Predictor Variables and Time 6 Community Participation Frequency and Variety

Pearson's correlations revealed small to medium associations between Time 6 community participation frequency and childhood autism symptoms (DBC2‐P ASA) (*r* = −0.36, *p* < 0.001) and Time 1 Self‐Absorbed behaviour problems (DBC2‐P) (*r* = −0.27, *p* < 0.05). Significant differences were found between Time 1 levels of degree of intellectual disability and community participation frequency [*F*(3,78) = 2.84, *p* < 0.05], however, post hoc analyses revealed no significant bivariate relationships. Only childhood autism symptoms and Time 1 Self‐Absorbed behaviour problems were associated with Time 6 variety of community participation activities (*r* = −0.28 and−0.24, respectively, both *p* < 0.05), although these correlations were small.

Multiple regression analyses were conducted to further explore the relative contribution of the significantly associated Time 1 variables on Time 6 community participation frequency and variety outcome (see Table 3). Time 1 degree of intellectual disability, autism symptoms (DBC2‐P ASA), and Self‐Absorbed behaviour (DBC2‐P) explained 13% of the variance in Time 6 community participation frequency. For Time 6 community participation variety, Time 1 autism symptoms and Self‐Absorbed behaviours explained 6% of the variance. Although both of the overall models were significant, none of the predictors contributed significantly to the outcome variable.

**TABLE 3:** Multiple regression analyses for the association between Time 1 degree of intellectual disability, behaviour and emotional problems, and autism symptoms, and Time 6 community participation frequency and variety.

Variable	*B*	SE *B*	*β*	*p*
Model 1: Community participation frequency (*n* = 82)				
Degree of intellectual disability^a^				
Severe/profound	−4.56	3.18	0.17	0.16
Mild	3.56	2.88	0.14	0.22
Average/borderline	0.69	2.49	0.03	0.78
Autism symptoms^b^	−0.37	0.20	−0.38	0.07
DBC2‐P Self‐Absorbed	2.89	6.19	0.10	0.64
Adjusted *R* ^2^	0.13			
*F*	3.34**			
Model 2: Community participation variety (*n* = 82)				
Autism symptoms^b^	−0.06	0.06	−0.20	0.36
DBC2‐P Self‐Absorbed MIS	−0.83	1.75	−0.10	0.64
Adjusted *R* ^2^	0.06			
*F*	3.52*			

*Note:* Adjusted *R*‐squared values are reported due to the small sample size.

Abbreviation: DBC2‐P = Developmental Behaviour Checklist 2—Parent.

^a^ Reference category is *moderate intellectual disability*.

^b^ Autism symptoms are measured using the DBC2‐P Autism Screening Algorithm.

** *p* < 0.01.

* *p* < 0.05.

### 3.3 Associations Between Time 6 (Adulthood) Predictor Variables and Time 6 Community Participation Frequency and Variety

Correlations revealed significant, moderate associations between Time 6 community participation frequency and Time 6 socioeconomic disadvantage (*r* = 0.30), autism symptoms, specifically ADI‐R Social/Communication domain (*r* = −0.40) and behaviour and emotional problems, specifically Self‐Absorbed (*r* = −0.37) and Social Relating (*r* = −0.30) behaviour problems (DBC2‐A) (all *p* < 0.01). There was a significant difference between levels of Time 6 degree of intellectual disability on community participation frequency score [*F*(3, 78) = 7.34, *p* < 0.001]. Frequency of community participation was significantly less among participants with severe/profound intellectual disability (*M* = 11.87, SD = 7.44) than in those with moderate intellectual disability (*M* = 20.70, SD = 10.83) and no intellectual disability (*M* = 22.81, SD = 8.65). There was a significant difference between different living arrangements [*F*(2,79) = 10.57, *p* < 0.001], where participants living in supported accommodation had significantly fewer community activities (*M* = 13.22, SD = 6.64) than both those living with family (*M* = 19.89, SD = 10.38) and those living independently (*M* = 24.93, SD = 7.79). There was also a significant difference between daytime activities [*F*(2,79) = 5.10, *p* < 0.01], with adults in mainstream activities participating significantly more frequently (*M* = 23.14, SD = 9.09) than those in no activity (*M* = 13.00, SD = 8.54) or disability specific activities (*M* = 17.00, SD = 9.25). There was no significant difference on community participation frequency for those with or without additional mental health disorder diagnoses [*t*(75) = 1.12, *p* = 0.266], and no significant relationship between community participation frequency and age (*r* = −0.14, *p* = 0.221) or gender [*t*(80) = 1.10, *p* = 0.275].

There were small to moderate significant correlations between Time 6 community participation variety and Time 6 socioeconomic disadvantage (*r* = 0.31, *p* < 0.01), autism symptoms, specifically ADI‐R Social/Communication domain (*r* = −0.38, *p* < 0.01), and ADI‐R Restricted and Repetitive Behaviours domain (*r* = −0.27, *p* < 0.05), and behaviour and emotional problems, specifically Self‐Absorbed and Social Relating behaviour (DBC2‐A) (*r* = −0.40 and *r* = −0.34 respectively, both *p*'s < 0.01). There was a significant difference between levels of adulthood degree of intellectual disability on community participation variety score [*F*(3, 78) = 4.71, *p* < 0.01]. Participants with severe/profound intellectual disability participated in significantly fewer activities (*M* = 4.61, SD = 2.29) than those with moderate intellectual disability (*M* = 7.00, SD = 3.43) and no intellectual disability (*M* = 7.12, SD = 2.39). There was also a significant difference between Time 6 living arrangements [*F*(2,79) = 5.11, *p* < 0.01], with those living in supported accommodation participating in fewer activities (*M* = 5.22, SD = 2.30) than those living independently (*M* = 7.60, SD = 2.41). There was no significant difference in terms of community participation frequency for those in different daytime activities at Time 6 [*F*(2,79) = 2.49, *p* = 0.09], with or without additional mental health disorder diagnoses [*t*(75) = 0.42, *p* = 0.68], and no significant relationship between community participation frequency and age (*r* = −0.10, *p* = 0.37) or gender [*t*(80) = −0.83, *p* = 0.41].

Multiple regression was used to explore the contribution of each of the associated Time 6 variables on community participation outcome. When considering community participation frequency, 30% of the variance was explained by Time 6 degree of intellectual disability, socioeconomic disadvantage, Self‐Absorbed behaviour problems, Social Relating behaviour problems, living arrangements, and daytime activities (see Table 4). In the model, living in supported accommodation was the only significant predictor when the other variables were controlled for. Living in supported accommodation was associated with a decrease of participation in community activities. When assessing the variety of community participation at Time 6, 20% of the variance was explained by Time 6 degree of intellectual disability, socioeconomic disadvantage, Self‐Absorbed behaviour problems, Social Relating problems and living arrangements (see Table 4). None of the predictor variables were significant in the regression model.

**TABLE 4:** Multiple regression analyses for the association between Time 6 degree of intellectual disability, behaviour and emotional problems, socioeconomic disadvantage, living arrangements and daytime activity, and Time 6 community participation frequency and variety.

Variable	*B*	SE *B*	*β*	*p*
Model 1: Community participation frequency (*n* = 74)				
Degree of intellectual disability^a^				
Severe/profound	−0.61	3.92	−0.03	0.88
Moderate	3.32	3.51	0.15	0.35
Mild	−3.84	3.21	−0.15	0.24
Socioeconomic disadvantage	0.02	0.02	0.16	0.16
DBC2‐A Self‐Absorbed MIS	−0.22	0.15	−0.21	0.15
DBC2‐A Social Relating MIS	0.04	0.27	0.02	0.89
Living arrangements^b^				
Supported	−6.11	2.63	−0.31	0.02*
Independently	2.85	3.02	0.12	0.35
Daytime activity^c^				
None	−6.89	3.52	−0.22	0.06
Mainstream	0.64	3.16	0.03	0.84
Adjusted *R* ^2^	0.30			
*F*	4.12**			
Model 2: Community participation variety (*n* = 74)				
Degree of intellectual disability^a^				
Severe/profound	−0.22	1.17	−0.04	0.85
Moderate	1.08	0.96	0.17	0.27
Mild	0.27	0.99	0.04	0.79
Socioeconomic disadvantage	0.01	0.01	0.20	0.09
DBC2‐A Self‐Absorbed MIS	−0.05	0.05	−0.17	0.29
DBC2‐A Social Relating MIS	−0.11	0.08	−0.16	0.19
Living arrangements^b^				
Supported	−1.09	0.79	−0.19	0.17
Independently	0.08	0.92	0.01	0.93
Adjusted *R* ^2^	0.20			
*F*	3.25**			

*Note:* Adjusted *R*‐squared values are reported due to the small sample size.

Abbreviations: DBC2‐A = Developmental Behaviour Checklist 2—Adult; MIS = mean item score.

^a^ Reference category is *average/borderline*.

^b^ Reference category is *living with family*.

^c^ Reference category is *disability specific activity*.

** *p* < 0.01.

* *p* < 0.05.

The association between current adult autism symptoms (Time 6) and Time 6 community participation frequency and variety was assessed in separate regression models (Table 5). For community participation frequency, 15% of the variance was explained by the combined ADI‐R Social/Communication domain mean algorithm scores. A negative beta value indicated that greater difficulty with reciprocal social interaction and communication (i.e., higher scores on the Social/Communication domain) was associated with a decreased frequency of community participation. For community participation variety, 15% of the variance was explained by the mean algorithm scores of the ADI‐R Social/Communication and Restricted and Repetitive Behaviour domains (Table 5). The ADI‐R Social/Communication domain was the strongest predictor of community participation variety, with a negative beta value indicating that greater difficulty with reciprocal social interaction and communication was associated with decreased variety of community activities.

**TABLE 5:** Multiple regression demonstrating the association between Time 6 autism symptoms and Time 6 community participation frequency and variety.

Variable	*B*	SE *B*	*β*	*p*
Model 1: Community participation frequency (*n* = 69)^a^				
ADI‐R Social/Communication	−3.78	1.06	−0.40	0.001**
Adjusted *R* ^2^	0.15			
*F*	12.65**			
Model 2: Community participation variety (*n* = 69)				
ADI‐R Social/Communication	−0.92	0.33	−0.33	0.01*
ADI‐R Restricted and Repetitive Behaviour	−1.31	0.85	−0.18	0.13
Adjusted *R* ^2^	0.15			
*F*	6.82**			

*Note:* Adjusted *R*‐squared values are reported due to the small sample size.

^a^ ADI‐R Restricted and Repetitive Behaviour is not included in the ‘frequency’ regression model as there was no significant correlation between community participation and mean ADI‐R Restricted and Repetitive Behaviour domain algorithm score.

* *p* < 0.05.

** *p* < 0.01.

## 4 Discussion

This study aimed to explore how autistic adults are participating in their communities, and the childhood and adulthood factors that predict frequency and variety of participation. Results suggested that adults in the study were participating in some areas of the community more frequently than in others. For example, participation in essential activities, such as shopping, and in social activities (e.g., going out to a café or restaurant; taking trips out with family or friends) was higher than participation in more structured community events, such as going to sports events, the cinema or a concert or play. It may be that these kinds of activities require more planning and are more expensive, for example, purchasing tickets and planning transport, and are therefore more complicated for adults to attend. These results were consistent with a recent study of autistic adults (19% with co‐occurring intellectual disability), reporting that shopping, errands, going to a restaurant or café, and time with family and friends were the most commonly attended activities as well as the most highly rated as being important to the individual (Shea et al. 2021). Ensuring that the availability and access to community activities aligns with individual preferences for what activities are considered important should be considered both in future research and in practice when supporting autistic adults with community engagement.

The current study did not compare participation with other samples or populations. However, the overall ICI frequency and variety scores were comparable to studies including adults with autism and co‐occurring intellectual disability (Felce et al. 2011; Totsika et al. 2010), and variety scores reported by Ager et al. (2001) in a group of adults with intellectual disability moving into community‐based homes. These studies were all conducted in the UK, and participants had a wider age range than the current study, and were all living in community‐based housing with at least some staff support. In the present study, individuals who were living in similar supported accommodation, and those with severe/profound intellectual disability, had lower participation rates, for both frequency and variety than others in the current study and the UK studies. This may point to a lack of services or appropriate activities for adults with autism and more severe/profound intellectual disability as well as for adults living in supported accommodation in Australia, limiting the opportunities available for autistic adults with additional support needs to engage in the community. It is imperative that services ensure the needs of adults with these additional support needs and those living in supported accommodation environments are addressed when planning community engagement activities.

When asked whether adults usually participated in each activity independently or with support, it was evident that few adults engaged in the community on their own. Over half of the participants were reported to have attended all community activities with a parent or carer, highlighting the ongoing support required. This study did not, however, ask whether the individual would be able to participate in the activity without support, or whether there were additional barriers, such as access to transport or financial limitations, that were preventing more independent participation. It may be the case that many of the adults who reported always attending events or activities with a parent or carer may desire to do this independently yet lacked the required skills or resources to do so. Further, previous research has demonstrated that self‐determination, particularly in relation to choosing which types of social activities to engage in, is associated with greater quality of life for autistic adults (Kim 2019). As a result, future research should ensure adults are asked what they want to do in the community, whether they prefer to participate in activities independently or with support from others, and what skills, resources or supports they need to be able to do this.

Childhood autism symptoms and Self‐Absorbed behaviour problems were significantly associated with both community participation frequency and variety, and childhood degree of intellectual disability was significantly associated with community participation frequency. Self‐Absorbed behaviour problems as measured by the DBC2‐P includes a high proportion of autism symptom‐like behaviours (e.g., *likes to play with unusual objects, stares at lights/spinning objects*). It is therefore likely that the relationship between this subscale and overall community participation outcomes is broadly reflective of autism symptoms. Few studies have considered the influence of childhood autism symptoms on community participation in adulthood however, greater difficulty with communication and social interaction in childhood has been associated with decreased social and community participation for autistic children (Askari et al. 2015). The current study suggests that children with a greater number of autism symptoms may also be at greater risk for reduced time in community activities as adults. Providing additional supports to children who experience difficulty with social and communication in accessing and engaging in community activities may lead to an increase in their community participation as adults.

A number of adulthood factors, including degree of intellectual disability, socioeconomic disadvantage, autism symptoms, Self‐Absorbed and Social Relating behaviour problems, living arrangements and current daytime activity, had significant, small to moderate relationships with community participation frequency and variety. However, regression analyses revealed only living arrangement to be significant when including other variables in the model. In particular, adults living in supported accommodation had much lower frequency of community participation when compared to adults living independently or with family. This may be attributed to difficulties for staff working in supported accommodation in having the necessary time and resources to support each adult in their care to participate regularly in the community. Further, while daytime activity did not remain significant when controlling for living situation, it was clear that adults who attend disability specific daytime activities have significantly less frequency of participation when compared to adults in mainstream employment or education. While disability‐specific day programmes often involve going out into the community to attend events or participate in other community activities, this may be the only time that these adults are engaging in community‐based activities. Access to support outside of day programmes to attend activities in the community may be limited, particularly for adults who are also living in supported accommodation.

Further, there was a significant association between adult autism symptoms and community participation, with scores on the combined Social/Communication domain of the ADI‐R correlated with community participation frequency and variety. Scores on the Restricted and Repetitive Behaviours domain were only correlated with variety of activities, and variance in the community participation variety outcome was better explained by scores on the Social/Communication domain. Together, these results demonstrate the impact of difficulties with social interaction and communication on regular engagement in the community. It may be that adults who experience greater difficulty with social interaction and communication choose to engage in recreation and leisure activities that involve less interaction with other people, such as playing video games or participating in other solo hobbies. This experience of social isolation has been demonstrated in other studies, where young autistic adults engage in fewer social activities than young adults with other developmental disabilities (Orsmond et al. 2013). However, the barriers created by the community are also important to consider. For example, misperceptions of autistic adults may mean that activities within the wider community are less inclusive (Mitchell, Sheppard, and Cassidy 2021). Further, limited accessibility of resources, such as transport or funding, may also prevent autistic adults from participating in the community as much as they would like (Chan et al. 2021; Song et al. 2023). These environmental barriers are important for future research to explore. Song et al. (2021) reported that community participation activities that involve social interaction are a low priority for autistic adults however, they did not explore whether this was due to a lack of social interaction skills. The current study is the first to look specifically at the impact of difficulty with social interaction on community participation. This is an important area for research to continue to explore, as social skills supports may be an important step in increasing community participation.

Diagnosis of a mental health disorder in adulthood was not associated with community participation, in contrast to a recent study by Shea et al. (2021). However, more specific behavioural problems, including Self‐Absorbed and Social Relating behaviours, were. The impact of behaviour and emotional problems on community participation warrants further investigation. While there does appear to be some relationship between behaviour and emotional problems and community participation, the direction of the relationship is not clear. Do higher rates of behaviour and emotional problems, and additional mental health difficulties, result in decreased community participation, or does restricted community participation impact behaviour and emotional problems? While there is evidence that increasing time spent in community or leisure activities can support improvement of symptoms of depression for adults with intellectual disability (Jahoda et al. 2017), similar outcomes have not been established for adults with autism, with or without intellectual disability (Cameron et al. 2021).

The current study had some limitations. First, the sample size (*n* = 82) was quite small, which should be considered when interpreting results from regression analyses. Despite the small sample size, all assumptions of analyses conducted were met. Secondly, this study did not ask participants whether there were other activities they would like to participate in, and what kinds of supports or resources would allow them to do this. The measure used in this study to assess community participation (the Index of Community Involvement) does not include some more contemporary means of participation such as special interest groups (e.g., meeting with others to play sport or other games). As a result, while the measure does ask about participation in a ‘social club’, some instances of social or recreational community engagement may not have been captured, possibly underestimating the level of engagement for some participants. Further, while the results of this study demonstrate that nearly all the adults participated in at least some kind of community activity over the previous month, whether this participation was meaningful for the individual was not assessed. Adults may be attending certain community activities not because they are important or meaningful for them, but simply because they were organised for them. Recent research has started to explore with autistic adults what activities are important to them (Shea et al. 2021), and it is important for future research to continue to ask these questions to ensure adults are supported to participate in activities that are meaningful to them. Further, autistic people should be involved in the codesign of future research, to ensure research in this space aligns with the priorities of the autistic community.

The study had several strengths. Firstly, as a longitudinal study, the childhood factors that may impact on community participation were able to be investigated, something few studies have explored. While diagnostic criteria for autism has changed since the participants in this study were children, along with changes regarding access and availability to supports and resources for autistic people through childhood and adulthood, the findings from this study highlight the broader factors that need to be considered when supporting autistic adults engaging in the community. For example, supporting people experiencing social and communication difficulties is likely to improve participation in the community regardless of when, or if, a diagnosis has been received. Secondly, the study included participants with and without intellectual disability, resulting in information being available for individuals with a wide range of abilities. This is particularly important as so few studies include adults with co‐occurring intellectual disability, or when they do, fail to report on how degree of intellectual disability was determined (Cameron et al. 2021). Finally, the current study made use of both parent/carer and self‐report data, maximising the information available. Using self‐report data where possible is important in allowing adults to contribute their own thoughts and responses.

Overall, this study demonstrated that, while some adults with autism are quite active in participating in the community, many experience much more limited participation. Further, it was evident that difficulties with social interaction, both in childhood and in adulthood, were important factors in limiting community participation for the adults in this study. This points to a need for increasing social support in childhood and adulthood. Further, it is necessary to create opportunities and programmes for adults with more severe intellectual disability to increase their participation in the community, particularly for individuals also living in supported accommodation. While there is a need for research to continue to explore the overall mental health benefits of community participation, removing barriers and increasing community participation for autistic adults is a critical step in promoting greater wellbeing. Exploration of the wider environmental and societal factors impacting community participation, including accessibility of transport, funding and programmes, as well as the attitudes and perceptions towards autistic people from the wider community, is important for future research. Further understanding of these factors will enable programmes and resources to best target ways to support and facilitate community participation. This may be through improved supports for autistic adults in addition to increasing education within the community of ways to be more inclusive of people with autism, making community participation more accessible and inviting. Most importantly, it is essential that any research, interventions, programmes or resources, work directly with adults to ensure that their own community participation preferences are being met.

## Author Contributions

Conceptualisation: Kylie M. Gray, Lauren A. Cameron, Bruce J. Tonge, Patricia Howlin, Stewart L. Einfeld and Roger J. Stancliffe. Methodology: Kylie M. Gray, Bruce J. Tonge, Patricia Howlin, Stewart L. Einfeld and Roger J. Stancliffe. Funding acquisition: Kylie M. Gray, Bruce J. Tonge, Patricia Howlin, Stewart L. Einfeld and Roger J. Stancliffe. Data curation: Lauren A. Cameron. Formal analysis: Lauren A. Cameron and Kylie M. Gray. Supervision: Kylie M. Gray. Writing – original draft: Lauren A. Cameron and Kylie M. Gray. Writing – review and editing: Lauren A. Cameron, Kylie M. Gray, Bruce J. Tonge, Patricia Howlin, Stewart L. Einfeld and Roger J. Stancliffe.

## Ethics Statement

This research received ethical approval from Monash University Human Research Ethics Committee (CF15/1045‐2015000486).

## Consent

Informed consent was obtained from all participants in this study.

## Conflicts of Interest

Bruce Tonge, Stewart Einfeld, and Kylie Gray are authors of the Developmental Behaviour Checklist (DBC2) and related materials. All royalties received by the authors from the sale of the DBC2 materials are donated to the funding of ongoing research in intellectual and developmental disabilities. Lauren Cameron, Patricia Howlin, and Roger Stancliffe declare no conflicts of interest.

## Data Availability

The data that support the findings of this study are available on reasonable request from the corresponding author. The data are not publicly available due to privacy and ethical restrictions.

## Endnotes

^1^
Throughout this paper, we use a combination of ‘person‐first’ (e.g., adult with autism) and ‘identity‐first’ (e.g., autistic adult) language to reflect the various views on terminology within the autism community (see, for example, Buijsman, Begeer, and Scheeran 2022; Keating et al. 2023).
